# ‘Cell knife’ for cancer: the clinician’s perspective

**DOI:** 10.3389/fimmu.2025.1536355

**Published:** 2025-04-17

**Authors:** Zihan Zhou, Yunhao Chen, Yao Wang, Yafang Hong, Hongdan Guan, Fenghao Huang, Fenfang Fu, Xiaobo Li, Rong Zheng, Benhua Xu

**Affiliations:** ^1^ Department of Radiation Oncology, Fujian Medical University Union Hospital, Fuzhou, China; ^2^ Department of Radiation Oncology, The Affiliated Cancer Hospital of Nanjing Medical University and Jiangsu Cancer Hospital and Jiangsu Institute of Cancer Research, Nanjing, China; ^3^ Fujian Key Laboratory of Intelligent Imaging and Precision Radiotherapy for Tumors, Fujian Medical University, Fuzhou, Fujian, China; ^4^ Clinical Research Center for Radiology and Radiotherapy of Fujian Province (Digestive, Hematological and Breast Malignancies), Fuzhou, Fujian, China

**Keywords:** boron neutron capture therapy, radiobiology, clinical trials, immunotherapy, combined treatments

## Abstract

Boron Neutron Capture Therapy (BNCT), often referred to as the ‘cell knife,’ represents a binary, tumor-selective therapeutic modality that minimizes damage to surrounding healthy tissues. This review provides a comprehensive clinical perspective on BNCT, addressing the radiobiological mechanisms and summarizing related clinical trials, with a particular emphasis on glioma and head and neck cancers. Furthermore, the paper touches upon the synergistic potential of BNCT when integrated with other treatment modalities, such as proton and carbon ion radiotherapy, alternative neutron capture therapies, ultrasound, and immunotherapy. These combined approaches may offer promising avenues for future research, potentially enhancing the therapeutic index and expanding the applicability of BNCT in oncological practice.

## Graphical Abstract

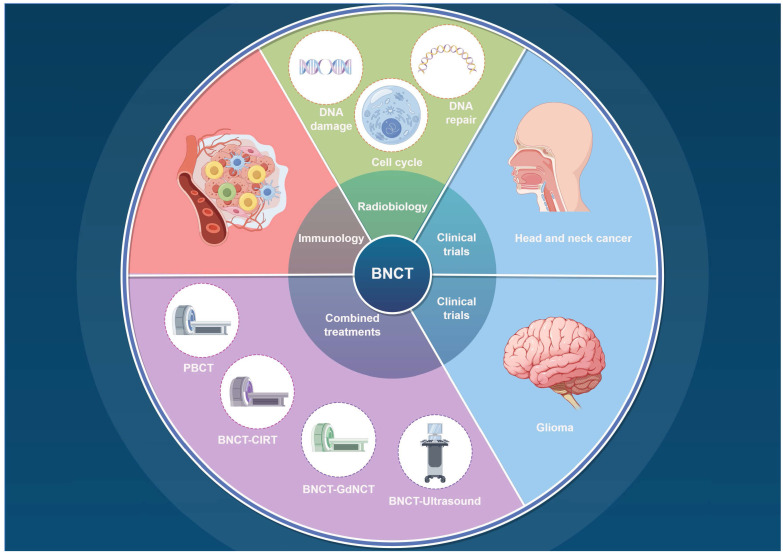

## Background

**Figure 1:**
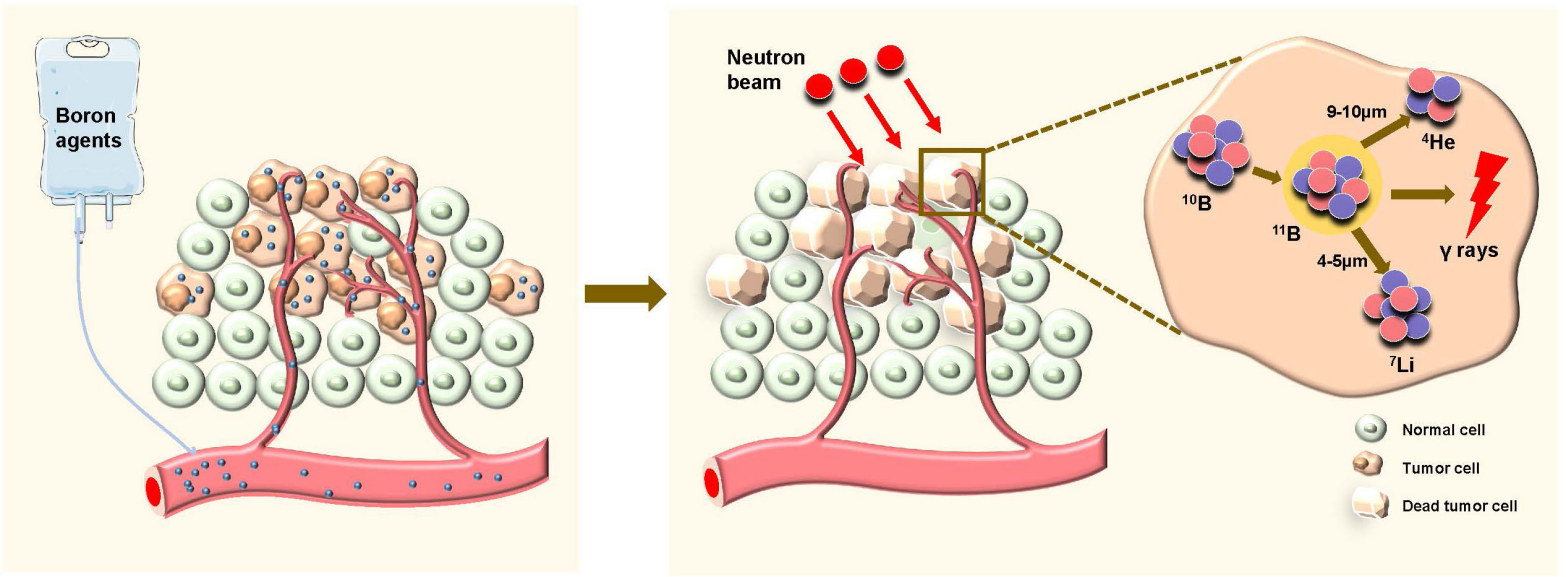
Schematic diagram of boron neutron capture therapy (BNCT) selectively killing tumor cells. Boron agents are administered and selectively accumulate in tumor cells. After the application of neutron beams, the neutrons are captured by boron-10 (¹^0^B) within the tumor cells, leading to the formation of helium-4 (^4^He) and lithium-7 (^7^Li), along with the emission of high-energy gamma rays. This reaction causes the death of the tumor cells while sparing the surrounding normal cells.

Boron neutron capture therapy (BNCT) is a highly accurate form of radiotherapy (RT) that combines targeted therapy with heavy ion RT. Ideally, nonradioactive ^10^B is taken up only by tumor cells. When ^10^B is irradiated with low-energy thermal neutrons, the unstable isotope ^11^B is created. Then, ^11^B undergoes instantaneous nuclear fission into recoiling ^7^Li nuclei and high-energy alpha particles (^4^He), which deposit their energies in the range of 5–9 µm (shorter than the cell diameter) (**Figure 1**). Hence, the harmful effects are limited to tumor cells (1). In recent years, monumental breakthroughs have been made in emerging methods of cancer treatment, such as targeted therapy, proton RT and heavy ion RT. However, several limitations and shortcomings remain. Targeted drugs kill tumor cells by targeting a link in the process of metabolism or proliferation. However, these links can be blocked or compensated for easily, resulting in drug resistance (2). Proton radiotherapy and heavy ion radiotherapy, high linear energy transfer (high-LET) methods, have shown significant cell-killing effects. These methods are more accurate than conventional RT according to the Bragg peaks. However, some healthy tissues are still exposed to radiation before the ray reaches the tumor. In addition, healthy tissues surrounding the tumor inevitably receive the same amount of irradiation (3).

At present, BNCT has entered the era of the accelerator, and new boron agents are being widely investigated. The development of imaging technology for dynamic monitoring has gradually increased. In 2020, permission for manufacturing and sailing accelerator-based BNCT equipment and boropharan was obtained from Japan for the first time (4). BNCT studies have since increased in countries such as America, Europe, Japan and China. In this review, the radiobiological mechanism of BNCT is introduced. The clinical results are summarized, with a focus on glioma and head and neck cancer (HNC). Perspectives on the combination of BNCT with other antitumor treatments are discussed. In addition, studies relevant to immunotherapy are presented.

## Treatment planning system and dose calculation

The treatment planning system (TPS) serves as the hub supporting the technology of BNCT. Compared with traditional photon or proton radiotherapy planning systems, BNCT-TPS faces three unique challenges: The first dimension is the complexity of the energy field, where neutron interactions with biological tissues result in secondary particle cascade reactions; the second dimension is pharmacokinetics, which involves accurately determining boron concentrations; and the third dimension is the specificity of biological effect calculations, which require the transformation of physical doses into biologically effective doses. The multi-physical field coupling characteristic of BNCT poses significant technical barriers in terms of algorithm innovation, data integration, and computational efficiency.

The planning module is the core of the TPS, which is based on the Monte Carlo method to calculate the dose distribution of patients in a mixed neutron-photon field (5, 6). The physical doses induced by neutrons consist of the boron doses produced by the ^10^B(n, α)^7^Li reaction, the nitrogen doses produced by the ^14^N(n, p)^14^C reaction, and the hydrogen doses produced by the ^1^H(n, n)p reaction (7). In BNCT, dose prescriptions refer to biological effective doses. Therefore, the planning module needs to perform calculations from neutron flux to physical dose to biological dose (while also considering the impact of γ rays in the mixed neutron-photon field and secondary γ rays produced by neutron interactions with biological tissues on the total biological dose). The calculated results are then returned to the TPS in the form of three-dimensional dose cloud maps and dose-volume histogram (DVH) diagrams. The formula for computing the physical dose is as follows. The absorbed dose Dn produced by the reaction of the neutron with each atom is given as follows:


Dn=∫t∫E fn(E)ϕ(E,t)dEdt

Where *f* is the factor releasing kinetic energy or dose conversion factor of photons in neutron matter, and *ϕ(t)* is the neutron flux or photon at a point. The value of *f* varies with the radiation energy. The dose component *D_woB_* is expressed as follows:


$$D_{woB}=D_{N}+D_{H}+D_{\gamma }$$

Where *D_woB_* is expressed as the sum of the nonboron dose components (8). *D_N_*, *D_H_*, and *D_γ_* are the nitrogen, hydrogen and γ dose components, respectively. The formula for calculating the biological dose is as follow:


$$\begin{array}{c} ED(Gy-Eq)=C_{B}\times D_{B,1ppm}\times CBE_{B}+D_{N}\times RBE_{N}+D_{H}\times \\ RBE_{H}+D_{\gamma }\times RBE_{\gamma } \end{array}$$

Where *ED* is equivalent dose. *C_B_* is the boron concentration. *D_B_* is the boron dose component. *CBE_B_* is compound biological effectiveness, which depends on the behavior of boron compound in each tissue. *RBE_N_*, *RBE_H_*, and *RBE_γ​_* are nitrogen, hydrogen, and γ absorbed doses of relative biological effectiveness, respectively.

## Radiobiological mechanisms

**Figure 2:**
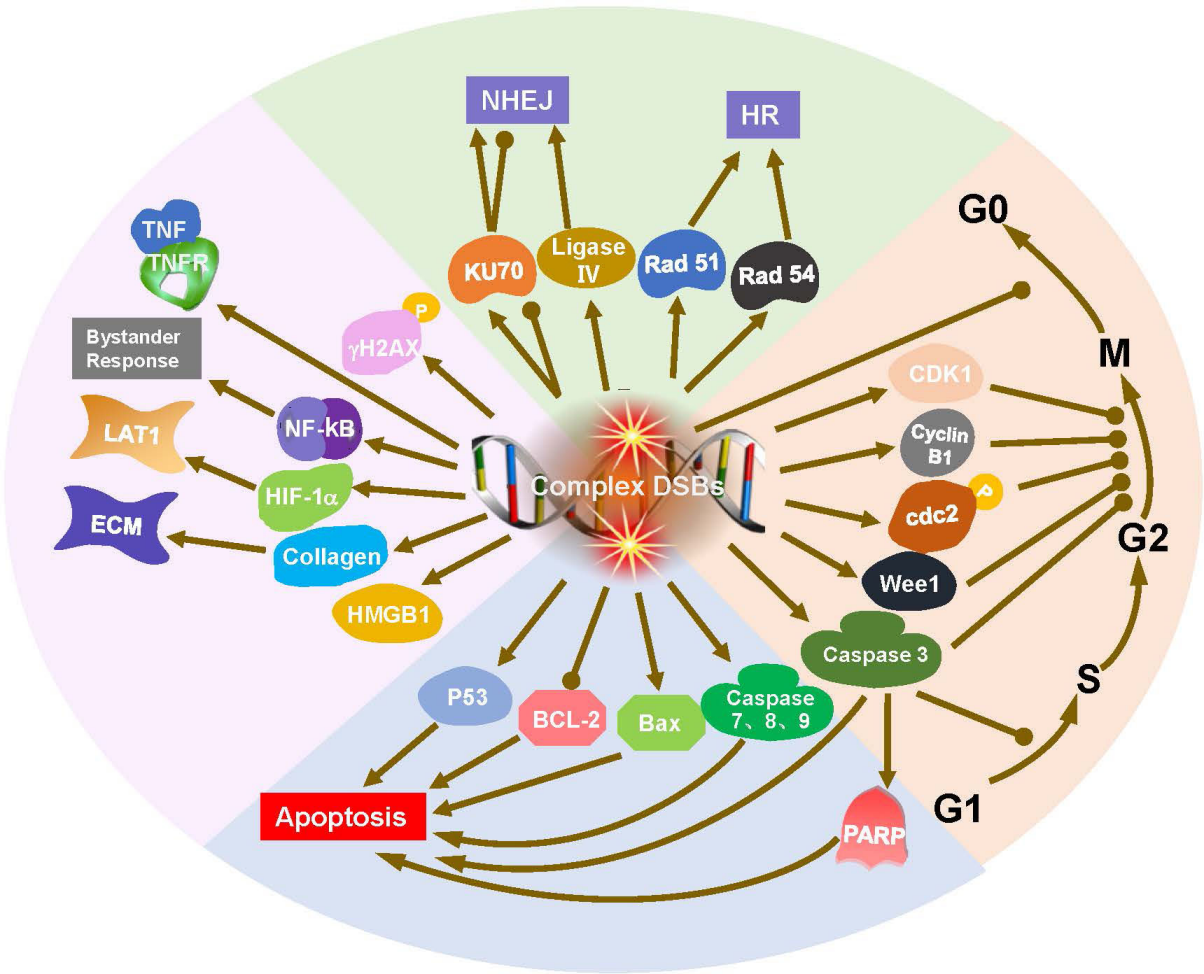
Radiobiological mechanisms of BNCT, including DNA damage, DNA repair, and cell cycle arrest and apoptosis. DNA double strand breaks (DSBs) initiate DNA repair, cell cycle arrest and apoptosis. DNA ligase IV and Ku70 are crucial for nonhomologous end joining (NHEJ), while Rad51 and Rad54 are integral to homologous recombination (HR). Cyclins and checkpoint proteins, such as Cyclin B1, CDK1, play important role in regulation of cell cycle. Bax activation and Bcl-2 downregulation are involvement in the apoptosis triggered by BNCT.

Ionizing radiation is characterized by its biological effects and is related to linear energy transfer. BNCT is a mixed-field irradiation technique comprising components with varying LET characteristics. These absorbed dose components are generally considered to act independently of each other (9, 10). Investigating the radiobiological mechanisms induced by BNCT will help researchers identify the cellular response markers and possible signaling pathways (**Figure 2**), thereby increasing therapeutic efficacy and reducing toxicity (11–13).

### DNA damage

DNA is the primary target of radiation damage, whether caused by phonons, protons, heavy ions, or neutrons (14, 15). γH2AX serves as a key marker for DNA double strand breaks (DSBs), initiating the recruitment of DNA repair proteins and playing a crucial role in maintaining genomic stability after irradiation (16). Masutani et al. reported an increase in γH2AX at 6 h after BNCT in a lymphosarcoma model. γH2AX and poly (ADP-ribosylation) (PAR) staining persisted at 20 h after BNCT (17). DNA damage increases with increasing radiation LET (18). High-LET particle components principally induce direct damage to DNA, causing irreparable DSBs, referred to as ‘complex DSBs’. Low-LET radiation primarily causes indirect, reparable DNA single-strand breaks (SSBs) (1, 14, 19–21). Epithermal neutrons can cause more than 50% of the DNA strands to break, and with increasing ^10^B concentration, more DNA strands break. Compared with photon RT, BNCT produces larger and more complex micronuclei in tumor cells (22, 23). BNCT produced a significantly larger focus size of γH2AX than phonon treatment did in a thyroid follicular cancer cell line (12).

### DNA repair

DNA damage activates the DNA repair system (24). In mammalian species, DSBs are repaired through nonhomologous end joining (NHEJ) in most cases. Natsuko Kondo et al. reported that BNCT-induced DNA damage can be partially repaired by the NHEJ repair protein DNA ligase IV (25). Ku70 is crucial for NHEJ, a faster but less accurate repair pathway primarily active in the G1 phase. In contrast, Rad51 and Rad54 are integral to homologous recombination (HR), a high-fidelity repair mechanism active in the S and G2 phases of the cell cycle (12, 14). Rodriguez et al. reported that the mRNA expression of Rad51 and Rad54 increased, but that of Ku70 did not significantly change (12). Perona et al. reported that Ku70 expression increased at different times after irradiation with neutrons but decreased after BNCT (neutrons plus BOPP). This decrease in Ku70 expression after BNCT explains the increase in sensitization to radiation in the BNCT treatment group (26).

### Cell cycle arrest and apoptosis

DNA damage naturally initiates cell cycle checkpoints, which provides cells with the time required for repair or to decide on programmed cell death if damage is irreparable (1, 27, 28). Cell cycle analysis revealed that BNCT induced G2/M arrest at 24 and 48 hours after irradiation (26). G2/M arrest has been associated with specific regulatory cyclin B1 (proteins associated with G2 arrest), and an inhibition or a delay in the activation of CDK1 (29, 30). Similarly, Sun et al. found a decreased expression of cyclin B1 and CDK1 proteins after BNCT (31). Fujita et al. and Kamida et al. also reported that Wee1, cdc2, and cyclin B1 were altered in the oral squamous cell carcinoma (OSCC) cell line SAS. Caspase 3 induces both G1 and G2 arrest, whereas apoptosis is related to G1 arrest (18, 32). BNCT inhibits OSCC cells in both p53-dependent and p53-independent manners. P53 is necessary for G1 arrest-associated apoptosis (1, 18, 32, 33). However, Seki et al. reported that DNA damage induced by BNCT was not dependent on p53 function (33). Wang et al. reported that the BNCT-induced apoptosis of glioma cells may be associated with Bax activation and Bcl-2 downregulation (34). However, Aromando et al. suggested that apoptosis may not play a significant role in BNCT-induced tumor control (35). To date, the role of apoptotic machinery after BNCT is still being explored.

Cells in S/G2/M phase having higher uptake of boronophenylalanine (BPA) than that in the G1/S phase due to metabolic activity (36). This effect was stronger with BPA than with borocaptate sodium (BSH), as BPA relies on cellular uptake, whereas BSH is a diffusion drug (36–38). Hypoxia-inducible factor 1α (HIF-1α) mediates adaptive responses to hypoxia and controls L-type amino acid transporter (LAT1) expression in hypoxic tumor cells (39, 40). In a lymphosarcoma model, high mobility group box-1 (HMGB1) levels increased 6 hours after BNCT and decreased at 20 hours (17). Unexpectedly, BNCT increased the metastatic potential of high-grade gliomas. This effect occurs because of bystander effects in adjacent cells; that is, BNCT can induce mutations in normal cells near boron-containing tumor cells, and NF-κB may be involved in the response (41). BNCT altered the extracellular matrix by decreasing collagen synthesis and elevated the levels of the tumor necrosis factor (TNF) receptor and cleaved caspases 3, 7, 8 and 9 in melanoma. These findings suggest that multiple pathways associated with cell cycle arrest and apoptosis are involved in the treatment of tumors by BNCT (42).

## Clinical studies of BNCT

**Figure 3:**
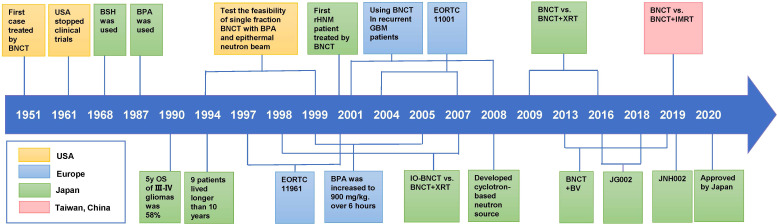
Historical timeline of landmark clinical studies on BNCT.

Recently, BNCT has been successfully used to treat high-grade gliomas and recurrent/metastatic HNC. Furthermore, it has potential for treating melanoma, breast cancer, angiosarcoma, etc (43). We summarize the clinical trials in which BNCT was used to treat patients with high-grade gliomas and HNC since 1994 in **Tables 1** and **2** and present the landmark studies in **Figure 3**. In addition, the registered trials are summarized in **Table 3**.

**Table 1:** Clinical trials of BNCT in glioma.

Country	Phase	Patients	Dates	Boron agents	Dose	Clinical outcome	Code	Ref
USA	I/II	53 GBM	1994-1999	BPA	The maximum dose in tumor volume ranged from 47.6-64.4 (mean 52.8 ± 4.2 Gy-Eq). The minimum dose in tumor volume ranged from 19.8 to 32.3 Gy-Eq (mean 25.2 ± 4.2 Gy-Eq).	MST: 12.8 mos. 2y OS: 9.4%	US-FDA IND #43,317	(44) (45)
USA	I	20 GBM 2 IC MM	1996-1999	BPA	Average tumor dose was estimated to range from 14.5 to 43.9 RBE Gy, with a mean of 25.7 RBE Gy.	MST: 11.1 mos (n=18) 2y OS: 12%		(46)
USA	I/II	6 GBM	2002-2003	BPA	Estimates of average tumor doses ranged from 33.7 to 83.4 RBE Gy (median 57.8 RBE Gy).	NA		(47)
European Organization	I	26 GBM	1997-2002	BSH	/	MST: 10.4-13.2 mos.	EORTC 11961	(48) (49) (50)
Finland		30 GBM	1999-2001	BPA	The average planning target dose was 25-29 Gy (W, W means radiobiologically weighted dose).	MST: 11.0-21.9 mos.	NCT00115453	(51)
Finland	I	20 rGBM 2 rA	2001-2008	BPA	The median average weighted PTV dose was 34 Gy (W), and the median average gross tumor dose was 38 Gy (W).	MST: 7 mos. post BNCT 1y OS: 36% 2y OS: 0%	NCT00115440	(52)
Czech		5 GBM	2000-2002	BSH	/	NA		
Sweden	II	29 GBM	2001-2003	BPA	The minimal weighted absorbed dose delivered to the tumor and target volumes ranged from 15.5 to 54.3 Gy and from 8.8 to 30.5 Gy.	MST: 14.2 mos. post BNCT 2y OS: 4/29 (13.8%)		(53)
Sweden		12 rGBM	2001-2005	BPA	Minimum tumor doses were in the range 13–27 Gy-Eq (median, 20).	MST: 8.7 mos. post BNCT		(54)
Japan		5 ndGBM 1 rGBM	1998-2000	BSH IO-BNCT	GTVmax: 20.0 ± 3.8 Gy; GTVmin: 18.0 ± 2.4 Gy; GTVmean: 19.3 ± 2.9 Gy.	MT: 15.5 mos. 2y OS: 0%		(55) (56) (57)
Japan		5 GBM 4 A	1999-2002	BSH IO-BNCT	The minimum boron dose for the tumor and target volume averaged 15.9 Gy (range 7.5–24.6 Gy) and 7.3 Gy (range 3.7–11.9 Gy)	MST: 23.2 mos. (GBM) MST: 25.9 mos. (AA)		(58)
Japan		7 GBM	1998-2007	BSH IO-BNCT	The minimal tumor dose for GTV was 16.3 to 63.0 Gy-Eq.	MST: 23.3 mos. 2y OS: 3/7 (42.9%)		(59)
Japan		8 GBM	1998-2007	BSH BNCT+XRT	The minimal tumor dose for GTV was 26.9 to 65.4 Gy-Eq.	MST: 27.1 mos. 2y OS:5/8 (62.5%)		(59)
Japan		10 ndGBM 1 rGBM	2001-2004	BSH IO-BNCT	GTVmax: 27.0 ± 7.3 Gy; GTVmin: 20.5 ± 5.3 Gy; GTVmean: 24.6 ± 5.3 Gy.	MST: 19.5 mos. 2y OS: 27.3%		(55) (56) (57)
Japan		3 ndGBM 7 rGBM	2002-2003	BSH+BPA	The minimal tumor doses for contrast-enhanced lesions ranged from 11.4 to 38.4 Gy-Eq.	MST: 14.5 mos. 2y OS:2/10 (20%)		(60) (61)
Japan		11 GBM	2003-2006	BSH +BPA BNCT+XRT	The minimal tumor doses for GTV in protocols 1 and 2 were 16.3 to 63.0 Gy-Eq and 26.9 to 65.4 Gy-Eq.	MST: 23.5 mos. 2y OS: 3/11 (27.3%)		(61)
Japan		19 rGBM 2 rA 1 rOA	2002-2007	BSH +BPA	/	MST: 10.8 mos. post BNCT 2y OS: 3/22 (13.6%)		(62)
Japan		6 GBM	2005-2008	BSH+BPA BNCT+XRT	The minimal physical and weighted dose of GTV in NO-BNCT were 7.8 ± 2.5 Gy and 27.7 ± 8.7 Gy (W).	MST: 26.2 mos. 2y OS: 50%		(56) (57)
Japan	II	21 ndGBM	2009-2016	BSH+BPA	Prescription dose by BNCT is regulated as not to be more than 13Gy-Eq for normal brain. Additional XRT is given with 3 gradient such as 8, 16, and 24Gy from the surface of scalp to the bottom of tumor infiltrated zone.	MST: 15.6 mos (all) MST:23.5mos.(BNCT+XRT) MST:14.1mos (BNCT)		(63)
Japan		14 pGBM 11 npGBM	2013-2019	BPA	The median values of maximum and minimum tumor doses were 75.6 Gy-equivalent (range: 35.9-151) and 39.4 Gy equivalent (range: 16.0-83.1).	MST: 21.4 mos. (pGBM) MST: 73.6 mos. (npGBM) 1y OS: 63.5% (pGBM) 1y OS: 81.8% (npGBM)		(64) (65)
Japan	II	27 rMG (24 rGBM)	2016-2018	BPA	The median of minimum tumor dose as a single fraction: 39.8 Gy-Eq (range: 23.1-63.2).	MST: 18.7 mos. 2y OS: 79.2%	JG002	(66)
Taiwan, China		15 GBM 4 BMG 11 A 2 OA 2 M	2017-2019	BPA	The mean tumor dose was 17.44 ± 7.50 Gy-E (mean physical dose = 5.75 ± 2.29 Gy).	MST: 7.25 mos. 1y OS: 29%		(47)

rGBM, Recurrence GBM; npGBM, non-primary glioblastoma; MG, glioma; A, Astrocytoma; OA, Oligoastrocytoma; BMG, Brainstem glioma; M, Medulloblastoma; RBE, Relative biological effectiveness; GTV, Gross tumor volume; PTV, Planning target volume.

**Table 2:** Clinical trials of BNCT in head and neck cancer.

Country	Phase	Patients	Dates	Boron agents	Dose	Clinical outcome	Code	Ref
Finland	I/II	12 rHNC	2003-2005	BPA	The median average tumor dose delivered to the GTV during the first scheduled BNCT was 21 Gy (W) (range, 14-29 Gy [W]) and during the second BNCT treatment 20 Gy (W) (range, 15-24 Gy [W]).	3 PR (25%) 7 CR (58.3%) 2 SD (16.7) 1y OS: 66.7%	NCT00114790	(67)
Finland	I/II	24 rSCC 6 rnSCC	2003-2008	BPA	The median calculated average tumor dose delivered to the GTV during the first scheduled BNCT was 23 Gy (W) [range, 14-37 Gy (W)], and during the second BNCT, it was 22 Gy (W) [range, 15-30 Gy (W)].	PR: 31% CR: 45% MST: 13 mos. post BNCT 2y OS: 30%	NCT00114790	(68) (69) (51)
Finland		6 rLC 3 LC	2006-2012	BPA	The estimated average GTV dose ranged from 22 to 38 Gy (W) (mean; 29Gy [W]).	MST: 13.3 mos. post BNCT		(70)
Finland	I/II	17 rHNC	2009-2013	BPA	/	NA (The neutron facility closed down for financial reasons)	NCT00927147	
German	I	6 SCC	2004-2007	3 BSH 3 BPA	/		EORTC 11001	(71) (72)
Japan		26 rHNC	2001-2007	BSH+BPA or BPA	The dose in deepest tumor of each patient was listed in Tables of the references.	MST: 7.9 mos. post BNCT PR: 38.5% CR: 46.2% 2y OS: 37%,		(73) (74)
Japan		49 rHNC 13ndHNC	2001-2007	BSH+BPA	The median minimum tumor dose was 17.9 Gy-Eq (range, 4.0-44.5 Gy-Eq).	PR: 29% CR: 28% MST: 10.1 mos. (n=53) 2y OS: 24.2%		(75)
Japan		2 rHNC 3 nd T4	2003-2007	BPA	The minimum GTV dose of 20 Gy-Eq was achieved in all patients, with the mean dose ranging from 32.9 to 82.3 Gy-Eq.	MST: 32 mos.		(76)
Japan		10 rSCC 7 rnSCC 3 nSCC	2003-2007	BPA	The control dose to the tumor is planned to be more than 20Gy-Eq (weighted dose).	PR: 35% 7 rnSqCC 3 nSqCC CR: 55% 2y OS: 32.3%		(77)
Japan		6 rOC	2005-2008	BPA	The maximum dose to the tumor (Gy-Eq) was 20.1-39.1 Gy-Eq and the minimum 9.12-31.9 Gy-Eq.	PR: 67% CR: 17%		(78)
Japan		7 SGC 4 sarcomas	2001-2012	BSH+BPA or BPA	/	MST: 24.2 mos. 5y OS: 50% (sarcomas) 5y OS: 38% (sSGC)		(79)
Japan		20 rSCC 8 MM	2012-2016	BPA	/			(80)
Japan	II	8 rSCC 13 R/LAnSCC	2019	Borofalan	The median tumor mean and minimum dose were 44.7 Gy-Eq (interquartile range, 42.9–50.6 Gy-Eq) and 31.1 Gy-Eq (interquartile range, 26.1-34.3 Gy-Eq).	2y OS: 58% (rSCC) 2y OS: 100% (r/LA nSCC)	JHN002	(81)
Japan		47rHNC	2020-2021	Borofalan	The minimum dose given to the GTV, of tumors was 27.4 Gy-Eq (range, 13.3-45.2 Gy-Eq; interquartile range, 24.6-31.0).	1y OS: 86.1% 2y OS: 66.5%		(82)
Japan		Recurrent and second primary cases: 25 HPC 11 LC	2020-2022		/	CR: 72% MST: 15.5 mos. 2y OS: 79.8%		(83)
Taiwan, China	I/II	12 rHNC	2010-2012	BPA	The first fraction of average equivalent GTV dose was 30.8 (26.0-39.6) Gy-Eq. The second fraction of average equivalent GTV dose was 15.1 (14.5-19.2) Gy-Eq.		NCT01173172	(84)
Taiwan, China	I/II	17 rHNC	2010-2013	BPA	For the GTV, the median D80 was 19.8 Gy-Eq (range, 6.7-37 Gy-Eq) and 14.6 Gy-Eq (range, 3.8-21.7 Gy-Eq) for the first and second fractions of BNCT.			(85)
Taiwan, China		9 rHNC	2019		The mean doses of GTV in BNCT and BNCT+IMRT plans were 23.52 ± 4.66 Gy (W) and 69.03 ± 1.56 Gy (W).			(86)
Taiwan, China	I/II	14 rHNC	2014-2022	BPA	The median BNCT average dose for the GTV was 21.6 Gy-Eq (range: 10.7–32.3 Gy-Eq).	CR: 35.7% PR: 28.6% 1y OS: 56%	IRB number 2012-06-016A	(87)

rHNC, recurrent head neck cancer; ndHNC, newly diagnosed head neck cancer; SCC, Squamous cell carcinoma; rSCC, recurrent squamous cell carcinoma; nSCC, non-squamous cell carcinoma; rnSCC, recurrent non-squamous cell carcinoma; LC, laryngeal cancer; rLC, recurrent laryngeal cancer; HPC, hypopharyngeal cancer; rOC, recurrent oral cancer; R/LAnSCC, recurrent/locally advanced; SGC, salivary gland cancer; GTV, Gross tumor volume; IMRT, Intensity-modulated radiation therapy.

**Table 3:** Clinical studies of BNCT(ICTRP) https://trialsearch.who.int/.

Tumor type	Country	Number	Date of Registration	Recruitment status
High-grade meninigioma	Japan	JPRN-jRCT2051190044	2019-09-04	Not Recruiting
Glioblastoma	Finland	NCT00115453; BNCT-P01	2005-06-22	Terminated
Newly Diagnosed Glioblastoma Multiforme	Japan	NCT00974987; OSAKA-TRIBRAIN0902; CDR0000650829; UMIN000002385	2009-09-01	Completed
Newly-diagnosed malignant glioma	Japan	JPRN-UMIN000003984	2010-08-03	Recruiting
Glioblastoma Multiforme And Intracranial Melanoma	Israel	NCT00039572; BIDMC-E-010284FB; CDR0000069398	2002-06-06	Completed
Glioblastoma Multiforme Removed During Surgery	Europe	NCT00004015; EORTC-11961	1999-11-01	Completed
Glioblastoma or Anaplastic Astrocytoma Progressing After Conventional External Beam Radiotherapy	Finland	NCT00115440; FIN-BNCT-03/2000; BNCT P-03	2005-06-22	Completed
Recurrent malignant glioma	Japan	JPRN-jRCTs051220019	2022-04-28	Recruiting
Recurrent malignant glioma	Japan	JPRN-UMIN000013419	2014-03-14	Pending
Recurrent malignant glioma	Japan	JPRN-jRCT2051210053	2021-07-16	Completed
Recurrent glioma	Japan	JPRN-jRCTs051180218	2019-03-27	Completed
Recurrent glioma	Japan	JPRN-UMIN000029144	2017-10-10	Recruiting
High-grade glioma	Japan	JPRN-C000000298	2006-04-01	Completed
Recurrent High-grade Gliomas	Korea	NCT05737212; DM-BNCT-P001	2023-02-09	Terminated
Recurrent malignant brain tumor	Japan	JPRN-UMIN000003692	2010-06-01	Completed
Primary malignant brain tumor and recurrent malignant head and neck tumors	Xiamen, China	ChiCTR2300078618	2023-12-14	Pending
Locally Recurrent Head and Neck Cancer	Taiwan, China	NCT01173172; BNCT_090514	2010-07-28	Completed
Locally Recurrent Head and Neck Cancer	Taiwan, China	NCT02004795; 2012-06-016A	2013-11-11	Recruiting
Locally Recurred Head and Neck Cancer	Finland	NCT00927147; HN-BPA-01-2008	2009-06-21	Terminated
Inoperable and Irradiated Head and Neck Tumors	Finland	NCT00114790; HN-BPA-01-2003	2005-06-17	Completed
Locally recurred head and neck cancer	Europe	EUCTR2008-004751-30-FI	2008-07-22	Not Recruiting
Relapsed and refractory head and neck malignancies or primary brain malignancies	China	ChiCTR2200066473	2022-12-06	Pending
Recurrent head and neck malignancies	Xiamen, China	ChiCTR2400082903	2024-04-10	Pending
Squamous cell carcinoma of the head and neck refractory to standard treatments	Japan	JPRN-UMIN000044118	2021-05-10	Pending
Head and neck malignancies	Japan	JPRN-UMIN000027543	2017-08-01	Completed
Recurrent and advanced head and neck cancer	Japan	JPRN-UMIN000011221	2013-10-01	Recruiting
Head and neck cancer	Korea	KCT0009158	2024-02-02	Recruiting
Melanoma	China	NCT02759536; XY3-IHNI1307A01	2016-04-18	Recruiting
Malignant melanoma	Japan	JPRN-UMIN000005124	2011-03-10	Completed
Melanoma (Skin)	US	NCT00059800; BIDMC-W-01-0380-FB; CDR0000287207; BIDMC-2001-P-001946	2003-05-06	Completed
Malignant Melanoma and Angiosarcoma (Skin)	Japan	NCT04293289; CNCT-001; SPM-011-JAM001	2020-02-25	Completed
Malignant Melanoma and Angiosarcoma	Japan	JPRN-UMIN000043564	2021-03-10	Completed
Malignant Melanoma and Angiosarcoma	Japan	JPRN-JapicCTI-195062	2019-12-03	Completed
Stage III Melanoma (Skin)	US	NCT00002781; CDR0000064811; NEDH-961207015; NCI-V96-0907	1999-11-01	Active, not recruiting
Metastatic Malignant Melanoma (Skin)	Europe	NCT00085059; EORTC-11011	2004-06-10	Terminated
Unresectable Angiosarcoma	Japan	NCT05601232; CNCT-002; SPM-011-JAM002; JPRN-jRCT2031220410	2022-10-26	Recruiting
Treatment-refractory angiosarcoma	Japan	JPRN-jRCTs051180217	2019-03-27	Completed
Refractory angiosarcoma	Japan	JPRN-UMIN000029401	2017-10-10	Pending
Skin malignant tumors	Japan	JPRN-UMIN000027541	2017-08-01	Completed
Brain tumor Head and neck tumor Other tumors (digestive organs, lung, skin, blood tumor etc.)	Japan	JPRN-UMIN000031323	2018-03-01	Pending
Recurrent Breast cancer	Japan	JPRN-jRCTs031220371	2022-10-07	Recruiting
Recurrent and refractory breast cancer	Japan	JPRN-jRCTs051180219	2019-03-27	Completed
Recurrent breast cancer	Japan	JPRN-UMIN000029403	2017-10-10	Pending
Malignant pleural mesotelioma	Japan	JPRN-UMIN000005478	2011-05-16	Recruiting
Recurrent advanced solid tumors	Xiamen, China	ChiCTR2400088140	2024-08-12	Pending

### Glioma

High-grade glioma is a category of aggressive primary brain tumors with limited therapeutic options and poor prognosis (88–90). Although glioblastoma (GBM) rarely metastasizes to other organs, it exhibits a highly infiltrative growth pattern (44). Traditional photon RT cannot kill infiltrating GBM cells, as the radiation dose required to eliminate tumor cells would also induce necrosis in the surrounding healthy brain tissue (44, 91, 92). Gliomas, account for most attempts to use BNCT in clinical settings, using the terminally differentiated nature of neurons to the advantage of a therapy based on lethal-upon-replication genome damage (13). GBM was chosen as the initial clinical target for phase I and II trials of BNCT (44). In 1951, Sweet and Javid reported the first case at the Brookhaven Graphite Research Reactor for primary brain cancer treated with BNCT (93). Forty brain tumor patients subsequently participated in the clinical trial. Unfortunately, patients experienced serious side effects, including scalp radiation damage, brain radionecrosis, cerebral edema and intractable shock, because the penetration force of the neutron sources used for treatment at the time was weak and the targeting of the boron agents was poor (94–99). Hence, the US completely discontinued clinical trials of BNCT in 1961. Hatanaka continued this research in Japan. In 1990, he reported that 120 patients with Grade III-IV gliomas whose tumors were within the limits of maximum therapeutic depth had a very satisfying 5-year survival rate of 58% (100). Four years later, he reported that 9 patients had lived longer than 10 years (101). This was an unexpected result and encouraged researchers to proceed with BNCT studies. In the 1990s, the USA initiated several clinical trials of BNCT with BPA and epithermal neutron beams (44–46). From September 1994 to May 1999, fifty-three primary GBM patients at the Brookhaven National Laboratory received BNCT after surgery via one, two or three irradiation fields. The median survival times (MST) were 14.8, 12.1 and 11.9 months, respectively. Extended exposure to thermal neutron beams was linked to increased neurotoxicity but was not positively correlated with improved local control or survival. This is indirect proof that BNCT has a greater advantage at low neutron irradiation doses (44, 45). The EORTC trial 11961 was launched in Germany in 1997 and included 26 GBM patients treated with BSH-based BNCT. This trial demonstrated the safety of BSH for clinical application at a dose rate of 1 mg/kg/min and a dose of 100 mg/kg. However, cerebral radiological changes, such as cerebral atrophy and white matter changes, appeared in half of the patients within the first year after BNCT (48–50). In the same period, intraoperative NCT and external beam NCT were compared in Japan. They found the MST of the two groups were 23.3 and 27.1 months, respectively (59). Twenty-two selected malignant glioma (MG) patients with progression after surgery and traditional RT entered a phase I study (NCT00115440) in Finland between 2001 and 2008. The MST after BNCT was 7 months, and the 1-year overall survival (OS) was 36% (52). In 2001, Sweden carried out two clinical studies in which the infusion of BPA was increased to 900 mg/kg body weight and was administered via a 6-hour intravenous infusion to increase the boron concentration in tumor cells. One study included 30 GBM patients, 27 of whom underwent debulking surgery, and reported that the boron concentration in the blood during irradiation ranged from 15.2-33.7 µg/g. Although the efficacy of BNCT was comparable to that of conventional photon RT, it worsened quality of life. Interestingly, patients treated with temozolomide (TMZ) at recurrence had a longer survival rate (17.7 months) than did those treated with BNCT alone (11.5 months) (53). The results of the other trial, which included 12 patients with recurrent GBM, were more encouraging. BNCT was reported to be as effective as concentration RT for recurrent GBM (54). Later, radiotherapy plus concomitant and adjuvant temozolomide was shown to be an optimal therapy for GBM (102). J.W. Hopewell et al. and Anja Sander et al. attempted to compare the OS between BNCT and RT+TMZ by reanalyzing the published data. Regrettably, no high-confidence results were found because of high patient heterogeneity across different trials (103, 104). Twenty-one newly diagnosed glioblastoma patients treated with BNCT had an MST of 15.6 months after diagnosis, which was significantly better than that of patients treated with postoperative radiotherapy and chemotherapy. There was a significant prolongation of survival in the BNCT+XRT boost group (MST, 23.5 months after diagnosis) compared with the BNCT alone group (MST, 14.1 months after diagnosis) (63). On the basis of these findings, a multicentric phase II clinical study was performed in Japan (NCT00974987), which planed enrolled 32 participants treated with BNCT, X-ray radiation treatment and TMZ. The outcome was not reported. In addition to TMZ, bevacizumab (BV) is an efficient antitumor drug for GBM. Twenty-five GBM patients with recurrent malignant glioma who were treated with BNCT and BV achieved prolonged OS and progression-free survival (PFS) compared to those achieved in prior BNCT-only studies. In addition, combining bevacizumab with BNCT may mitigate adverse effects such as pseudo-progression and radiation necrosis (64, 65). A multi-institutional, open-label, phase II clinical trial for 27 recurrent MG patients was conducted with the abovementioned accelerator-based BNCT system (JG002). BV-naïve MG patients who experienced recurrence after standard treatment were enrolled between February 2016 and June 2018. In that study, the 1-year survival rate was 79.2% and the MST was 18.7 months in recurrent GBM patients, whereas those of JO22506 patients were 34.5% and 10.5 months, respectively (https://meetings.asco.org/abstracts-presentations/190090). These results are exciting, but the monitoring efficacy of magnetic resonance imaging cannot provide sufficient information on the biological features of the tumor to identify pseudo-progression. Therefore, the use of ^18^F-BPA-PET to monitor treatment efficacy and evaluate patient prognosis is promising. If the trial is successful, recurrent MG may have a new indicated therapy (JPRN-UMIN000022850).

### Head and neck cancer

With successful clinical trials for the treatment of MG underway in the 1990s, researchers began to focus on other cancers. BNCT has achieved great success in treating HNC. Before the advent of immunotherapy, therapeutic approaches for recurrent or locally advanced HNC included only RT, platinum drugs and cetuximab. Thus, there is much room for the development of BNCT to treat HNC. The first patient with recurrent HNC received BNCT in 2001. In the 2000s, several clinical trials were performed in Finland. In a prospective, single-center phase I-II study (NCT00114790), twelve patients with locally advanced inoperable HNC were treated with BNCT. The outcomes included partial response (PR), complete response (CR) in 7 patients, and stable disease (SD) in 2 patients, and the 1-year OS was 66.7%. BNCT was thus shown to be an effective and safe treatment modality for locally advanced inoperable HNCs that recur at previously irradiated sites (67). Another clinical trial (NCT00114790) involving 30 patients with inoperable, locally recurrent HNC also utilized BNCT. The MST after BNCT was 13 months, and the 2-year OS was 30%. BNCT has shown efficacy in treating patients with cancer recurrence at previously irradiated sites, although recurrence remains common (51, 68, 69). The EORTC 11001 study explored the feasibility of BNCT for head and neck squamous cell carcinoma (HNSCC). Prior to the planned tumor resection, three patients received BSH, and three received BPA (71, 72). At that time, several trials were performed in Japan that combined BSH and BPA. There was a cohort of 62 patients, and primary severe Grade 3 or 4 toxicities were manageable (75, 81). In 2008, the dream of “from reactors to accelerators” came true. One interesting trial, including 9 patients with recurrent HNC, was performed in Taiwan, China, in the era of intensity-modulated radiation therapy (IMRT) and suggested that BNCT combined with compensated IMRT can increase treatment homogeneity and conformity compared with BNCT alone, particularly for tumor volumes exceeding 100 cm^3^, and may improve local tumor control (86). The JHN002 trial included patients with recurrent SCC or with recurrent/locally advanced non-SCC (R/LAnSCC). The ORR for all patients was 71%, and the 2-year OS rates for R-SCC and R/LAnSCC were 58% and 100%, respectively (81). Because of the success of JHN002, the Japanese government approved accelerator-based BNCT equipment, boropharan and health insurance coverage of BNCT for HNC in 2020 (4, 105). Under the Japan National Health Insurance System, a retrospective analysis investigated the first 47 patients treated with BNCT between May 2020 and February 2021 in Japan. All patients had undergone RT. The minimum dose administered to the tumor was 27.4 Gy-Eq, with a range of 13.3–45.2 Gy-Eq. The overall survival rates at 1 and 2 years were 86.1% and 66.5%, indicating high degrees of efficacy and safety (82). Similarly, the other retrospective study included 36 hypopharyngeal/laryngeal cancer patients with prior head and neck irradiation. The CR rate was 72%, and the objective response rate (ORR) was 84%. The MST was 15.5 months, and the 2-year OS was 79.8%. No acute G4–5 adverse events (AEs) were observed except for hyperamylasemia, and no late-phase G3 or higher AEs occurred. This finding demonstrates again that BNCT can achieve a good tumor response while preserving the larynx without severe AEs (83). In addition, a prospective phase I/II trial enrolled 14 patients, and 12 patients received combined treatment. The median BNCT average dose for the gross tumor volume (GTV) was 21.6 Gy-Eq, and the median image-guided intensity-modulated radiotherapy (IG-IMRT) dose for the PTV was 46.8 Gy/26 fractions. The 1-year OS and local PFS rates were 56% and 21%, respectively. Although the trial showed a high response rate (64%), it also experienced a significant incidence of in-field and marginal failure. Future research could explore combining BNCT with non-radiation modalities (87).

## Combined treatments

Dual-modality cancer treatment may synergistically enhance treatment efficacy. In recent years, the use of BNCT plus another therapeutic modality has gained increasing attention, although such approaches have yet to be widely adopted in clinical practice. Several combined regimens have been proposed, such as BNCT-photon (see clinical trials), BNCT-proton, BNCT-carbon ion radiotherapy (CIRT), BNCT-GdNCT, and BNCT-Ultrasound.

### Proton RT

Proton therapy is a promising radiotherapy modality for treating deep-seated and unresectable tumors. However, its biological advantages have not yet been addressed. In 2014, Do-Kun et al. first proposed the idea of proton-boron capture therapy (PBCT), a modality that combines the concepts of proton RT and BNCT (106). Four years later, Cirrone et al. experimentally tested this idea for the first time by using the p+^11^B→3α nuclear fusion reaction to increase the biological effectiveness of protons (107). Two subsequent reports demonstrated that osteosarcoma cells (108), prostate cancer cells and glioblastoma cells (109) treated with PBCT exhibited reduced survival and increased chromosomal aberrations compared with those treated with protons alone. Furthermore, Manandhar et al. first reported the use of DSBs as a surrogate measure of the dose enhancement effect of alpha particles arising from the proton–boron reaction. They discovered that BSH radiosensitized cells to protons, but this effect was independent of DNA damage (110).

### CIRT

CIRT is a therapeutic modality that relies on the Bragg peak of carbon ions to achieve precise and conformal dose deposition in tumors (111). Theoretically, the combined BNCT–CIRT modality can offer a more homogeneous tumor dose distribution and lower normal tissue toxicity by integrating the biological targeting capabilities of BNCT with the intensity modulation capabilities of CIRT. Han et al. assessed the feasibility and potential advantages of integrating BNCT with CIRT. BNCT–CIRT ensures uniform delivery within the clinical tumor volume (CTV) via the reversed gradient effect from the CIRT component. BNCT–CIRT can thus minimize damage to normal brain tissue and skin (112).

### GdNCT

GdNCT is another neutron capture therapy (NCT). BNCT and GdNCT have their own merits and limitations. Higher levels of DNA damage are caused by the release of secondary high-LET particles during BNCT, but improved dose uniformity is expected for the GdNCT technique because secondary particles with lower LET values (electrons and photons) are released during the GdNCT, and ^157^Gd has a higher neutron capture cross-section than ^10^B, allowing the use of a lower neutron flux for the NCT technique. In addition, the secondary particles released after neutron capture by ^157^Gd have long ranges inside the target volume. Therefore, a combination of ^10^B and ^157^Gd may improve treatment efficiency in terms of dose uniformity and the relative biological effectiveness (RBE) of DNA damage. Shamsabadi et al. reported that the combined Gd/BNCT technique increases tumor coverage at relatively high doses but reduces the RBE model of DSB induction, potentially affecting the clinical efficacy of NCT (113).

### Ultrasound

The mechanism of ultrasound differs from that of RT (direct DNA damage). First, ultrasound enhances the sensitivity of tumor cells to radiotherapy (114–116). Second, ultrasound with microbubbles is expected to increase BPA uptake in tumor cells, thereby increasing the boron adsorption capacity. Ultrasound-induced cavitation can compromise the integrity of endothelial and tumor cells by disrupting cell–cell junctions and causing leakage of transport molecules from blood vessels into the tumor microenvironment (TME) (117). Notably, previous study has shown that focused ultrasound (FUS), when combined with a microbubble agent, has ability to temporarily disrupt blood-brain barrier (118). Microbubble-based sonoporation has been shown to reduce the expression of P-glycoprotein in the blood-brain barrier in rats (119). This would increase drug penetration and accumulation in the central nervous system (120), which is one of the excellent advantages for the treatment of glioma using BNCT.

## BNCT and immunotherapy

It is now widely acknowledged that high-LET irradiation may be more immunogenic (121). BNCT combined with immunotherapies will thus be a natural future direction (**Table 4**). As early as 2000, Smilowitz et al. used a malignant rat glioma model of high immunogenicity to evaluate the efficacy of the combination of BNCT and immunoprophylaxis. Half of the rats died after treatment with BNCT alone, but survival was higher after combined treatment. Most surviving rats display immune memory six months or longer after treatment (122). Although second-generation boron agents, including BPA and BSH, have been put into clinical application, they are still not ideal because of insufficient tumor specificity. Synthetic, highly selective and safe boron delivery drugs constitute the key way to overcome the bottleneck of BNCT. Ali Khan et al. reported that the presence of boron-rich liposomes in the blood is crucial for the inhibitory effects of BNCT, whereas direct injection showed no additional benefit in a breast cancer BALB/c mouse model. Compared with other blood components, peripheral blood mononuclear cells (PBMCs) are more likely to assimilate boron-rich liposomes. However, irradiation did not damage the boron-carrying PBMCs. BNCT in PBMCs caused these cells to adopt an antitumor phenotype characterized by increased IL-12 and decreased IL-10 levels. These findings indicate that boron-rich liposome-based BNCT can increase antitumor immunomodulatory effects (123). Shi et al. engineered a neutron-activated boron capsule that synergizes BNCT and controlled immune adjuvant release to elicit a strong antitumor immune response. Like photon radiation, BNCT can remodel the tumor immune microenvironment. Single-cell RNA-Seq analysis indicated that PEG-B-COF+ neutron-treated tumors presented elevated levels of CD4+, CD8+, and Natural killer (NK) cells and a reduced proportion of myeloid cells. The expression of protumoral and immunosuppressive genes was downregulated, whereas the expression of proinflammatory chemokine genes, T/NK cell activation genes, and T/NK cell effector genes was upregulated. Moreover, BNCT can induce immunogenic cell death and exert an abscopal effect (125). Kinashi et al. reported that low-energy head-neutron irradiation damages immune organs in radiosensitive SCID and BALB/c mice and that the combination of BNCT and immunotherapy may not only increase the efficacy but also attenuate damage caused by BNCT (126).

**Table 4:** BNCT combined with immunotherapy.

Year	Tumors	Models	Boron agents	Dose	Immune types	Combination with immune drugs	Ref
2000	GBM	9LGS-Rat	1200mg BPA/kg		/	Immunoprophylaxis (a form of active immunization)	(122)
2019	Breast Cancer	BALB/c mice	BPA		PBMCs	TAC/MAC liposomes	(123)
2022	GBM	C57BL/6 mice	CB/DOX-CB@lipo-pDNA-iRGD	the ion source (1879 V, 0.208 mA, 90 kV, 1.58% × 10^8^/s)	/	DOX-CB@lipo-pDNA-iRGD (blocking macrophage immune checkpoint pathway CD47-SIRPα by CRISPR-Cas9 system)	(124)
2023	Melanoma, Colon carcinoma	B16F10, MC38-C57BL/6 Mice	1mg/mL B-COF	1.9 × 10^9^/(cm^2^·s) 10min	CD45+ cells	Imiquimod (toll-like receptor 7 agonist)	(125)
2023	–	Balb/c, SCID, C3H Mice	Kyoto University Research Reactor (KUR) and thermal neutron fluences	1-MW neutron beam; Thermal neutron fluences 2.3 ± 0.2 (E+12) cm^–2^; Physical dose 1.0 ± 0.1 Gy.	/	/	(126)
2023	HNSCC	C57BL/6 mice	350 mg/kg L-BPA (L-4-Boronophenylalanine, GHP-001)	1.2-MW epithermal neutron beam with a flux > 1.3 ×10^9^ n/cm^2^/s	MDSCs	CSF-1 receptor (CSF-1R), PLX3397	(127)
2023	Colon cancer	BDIX rats	BPA/Borophenylalanine+GB-10/Decahydrodecaborate	4.2 × 10^12^ n cm^−2^; 18-25 min		Oligo-Fucoidan (O-Fuco) or Glutamine (GLN)	(128)
2024	Melanoma	C57BL/6JNarl mice	mPEG-b-(PVB-r-PVBE) block copolymer	1 × 10^9^ neutrons/cm^2^·s; 1.2 MW, 30 min	–	PD-L1 antibody (B7H1)	(129)
2024	Melanoma	C57BL/6 mice	100 μg mL^−1^ boron nitride nanoparticles	2.5 kW, 2.57 × 108 cm^−2^·s^−1^; 3 h	CD4, CD8 T cells	BEV@BMDC	(130)
2024	Melanoma	C57BL/6J mice	500 mg/kg BPA	5 MW neutron irradiation	CD8+ T cells	PD-1 antibody (# BE0146)	(131)

Currently, PD‐1/PD‐L1 inhibitors are the most widely used for immunotherapy. Fujimoto et al. first demonstrated the abscopal effect induced by the combination of BNCT and an anti-PD-1 antibody in an immune checkpoint inhibitor (ICI)-resistant melanoma model (131). Almost simultaneously, Chiu et al. developed amphiphilic PEG-b-PVBE block copolymer micelles and combined these micelles with PD-L1 antibody treatment in a melanoma model. Compared with BNCT alone, combination therapy more effectively inhibited tumor growth and significantly increased T-cell infiltration and activation at tumor sites, indicating a stronger immune response (129).

Myeloid-derived suppressor cells (MDSCs) are a heterogeneous group consisting of granulocytic (G-MDSC) and monocytic (M-MDSC) subsets, each of which inhibits immune function through distinct mechanisms (132, 133). One study revealed that MDSC depletion (CSF-1R inhibitor) combined with BNCT extended mouse survival and promoted tumor immunity by reducing the number of tumor-associated macrophages and increasing the number of CD8+ T cells (127). CD47-blocking immunotherapy activates macrophage-mediated phagocytosis, increases adaptive immunity, and decreases the risk of recurrence. Chen et al. designed a multifunctional nanoliposome delivery system to transport a CD47 targeted CRISPR–Cas9 gene knockout plasmid and a boron delivery drug to the nucleus of tumor tissue in a GBM mouse model, thus increasing antitumor effectiveness (124). Dendritic cells (DCs) are optimal targets for delivering immunogenic cargo because of their strong antigen-presenting abilities. Recently, Lv et al. prepared BMDCs pulsed with BNCT-irradiated tumor cell-derived extracellular vesicles (BEVs) as a tumor vaccine candidate (named BEV@BMDCs), and this treatment elicited strong antitumor immunity *in vivo*. Vaccination with BEV@BMDCs suppressed primary tumor growth, prevented the formation of metastatic foci, and induced a long-lasting immune response (130).

In addition to being combined with immunotherapy, BNCT combined with anti-inflammatory and anticancer substances may elicit much stronger immune responses. Frydryk Benitez et al. combined (BPA/borophenylalanine+GB-10/decahydrodecaborate)-BNCT (Comb-BNCT) with oligo-fucoidan or glutamine in colon cancer models. They reported that, compared with BPA-BNCT, Comb-BNCT increased therapeutic efficacy, reduced radiotoxicity, and induced both an immune response and an abscopal effect (128).

## Discussion and conclusion

BNCT is a type of binary therapy for cancer treatment designed to address resistance to conventional treatment. BNCT has notable advantages for the treatment of multiple tiny metastatic foci, and the whole process requires only 1–2 treatments, saving cost and time. This review inspired us several directions for future study. First, understanding the essential molecular mechanisms of BNCT is important. However, mechanistic studies of events in the cell following BNCT are scarce, and most current considerations on this topic are largely inferred from information about the biological effects of high-LET radiation from other sources and of radiomimetic drugs. Therefore, this study could be helpful for researchers and practitioners to better understand the similarities between BNCT-induced damage and other types of radiation damage, as well as the cellular responses to this damage. Second, although clinical trials in glioma and HNC patients have achieved outstanding success, while a strong heterogeneity has been revealed among trials. No randomized controlled trials are currently comparing the first-line treatment of patients. Further optimization and well-designed randomized controlled trials are needed to further validate the efficacy and safety of BNCT in other cancer types. In addition, since BNCT is typically considered during local recurrence, it faces significant challenges in curing tumors during the initial treatment phase. It is important to standardize the treatment protocol of BNCT. Future studies should focus on standardizing treatment protocols and addressing limitations to guide clinical decision-making. Finally, a more profound understanding of the TME and increasingly mature BNCT techniques are needed. We to some extent fill the gap about the role of BNCT on the TME. Combined treatments, including photon, proton, CIRT, GdNCT, and ultrasound, may be new directions for future research. Thus, preclinical studies are needed to understand the radiobiological characteristics and immunomodulatory mechanisms of BNCT. BNCT has a long history, but comprehensive studies are subject to limitations because of the upper threshold level. In particular, BNCT is an intrinsically multidisciplinary field that requires cooperation from researchers in nuclear physics, chemistry, pharmacology, oncology, imaging, computer science and other areas. Additionally, the development of small BNCT devices based on miniaturized accelerators or small neutron sources, such as Cf-252 sources, are needed to reduce the treatment costs of BNCT.

## Glossary

AEs: adverse events
BEVs: BNCT radiated tumor cell-derived extracellular vesicles
BNCT: Boron neutron capture therapy
BPA: Boronophenylalanine
BSH: borocaptate sodium
BV: bevacizumab
CIRT: carbon ion radiotherapy
CR: complete response
CT: computed tomography
CTV: clinical tumor volume
DCs: Dendritic cells
DSBs: double strand breaks
DVH: dose-volume histogram
FUS: focused ultrasound
GBM: glioblastoma
Gd: gadolinium
GdNCT: Gd neutron capture therapy
G-MDSCs: granulocytic MDSCs
GTV: gross tumor volume
HIF-1α: Hypoxia-inducible factor 1α
HMGB1: high mobility group box-1
HNC: head-neck cancer
HNSCC: head and neck squamous cell carcinoma
HR: homologous recombination
ICI: immune checkpoint inhibitor
IMRT: intensity-modulated radiation therapy
LAT1: L-type amino acid transporters
^7^Li: lithium-7
LET: linear energy transfer
MDSCs: Myeloid-derived suppressor cells
MG: malignant glioma
M-MDSCs: monocytic MDSCs
MRI: magnetic resonance imaging
MST: Median survival times
NCT: neutron capture therapy
NHEJ: non-homologous end joining
NK: natural killer
ORR: objective response rate
OS: overall survival
OSCC: oral squamous cell carcinoma
PAR: Poly(ADP-ribosylation)
PBCT: proton-boron capture therapy
PBMCs: peripheral blood mononuclear cells
PFS: progression-free survival
PR: partial response
RBE: relative biological effectiveness
R/LAnSCC: recurrent/locally advanced non-SCC
RT: radiotherapy
SD: stable disease
SSBs: single-strand breaks
TME: tumor microenvironment
TMZ: temozolomide
TNF: tumor necrosis factor
TPS: treatment planning system
